# An Improved Tat/Rev Induced Limiting Dilution Assay With Enhanced Sensitivity and Breadth of Detection

**DOI:** 10.3389/fimmu.2021.715644

**Published:** 2021-08-05

**Authors:** Kavita Mehta, Yuvrajsinh Gohil, Swarnima Mishra, Anish D’silva, Afzal Amanullah, Deepak Selvam, Neelam Pargain, Narendra Nala, G. N. Sanjeeva, Udaykumar Ranga

**Affiliations:** ^1^Molecular Biology and Genetics Unit, Jawaharlal Nehru Centre for Advanced Scientific Research, Bengaluru, India; ^2^Department of Pediatric Genetics, Indira Gandhi Institute of Child Health, Bengaluru, India

**Keywords:** latent reservoir, HIV latency, HIV-1, TILDA, transcriptional silence

## Abstract

Tat/Rev Induced Limiting Dilution Assay (TILDA) is instrumental in estimating the size of latent reservoirs of HIV-1. Here, we report an optimized TILDA containing a broader detection range compared to the reported methods and high sensitivity. Giving priority to sequence conservation, we positioned the two forward primers and the probe in exon-1 of HIV-1. The reverse primers are positioned in highly conserved regions of exon-7. The optimized TILDA detected eight molecular clones belonging to five major genetic subtypes of HIV-1 with a comparable detection sensitivity. Using the optimized assay, we show that only a minor proportion of CD4^+^ T cells of primary clinical samples can spontaneously generate multiply spliced viral transcripts. A significantly larger proportion of the cells produced viral transcripts following activation. The optimized TILDA is suitable to characterize HIV-1 latent reservoirs and the therapeutic strategies intended to target the reservoir size.

## Introduction

Despite the considerable success of antiretroviral therapy (ART) in blocking HIV-1 replication, the virus persists in a small subset of cells where it remains transcriptionally silent (1, 2). The total repertoire of replication-competent and defective proviruses of an infected subject is called the latent reservoir (LR). Interruption of ART leads to re-emergence of the virus, even after years of undetectable viral load (3, 4). Despite successful ART, a low-level viral proliferation still occurs primarily due to the presence of LR; thus, the presence of LR offers a major challenge to disease management (5). LR ensures the continued presence of the provirus in a subject, thus, posing the major obstacle to a ‘functional’ cure. Thus, eliminating the LR becomes vital for HIV cure.

Accurate measurement of the viral reservoir size is crucial but challenging for at least two technical reasons. First, many integrated viral genomes contain inherent defects and cannot produce infectious viruses following activation. The viral sequences may contain debilitating mutations, deletions, or other defects in regulatory regions such as packaging signals, etc., resulting in the production of non-infectious virions (6, 7). Second, for reasons not yet completely understood, only a small fraction of replication-competent proviruses is activated following activation of any kind (8, 9). Ho et al. demonstrated that approximately only 12% of infected cells contain a replication-competent viral genome, while the remaining 88% of cells have large deletions making them defective (6).

Since the initial characterization of LR in the 1990s, several strategies have been developed for molecular characterization of the latent proviruses and reservoirs (10). The LR assays vary considerably in assay complexity, sensitivity, and the viral product they measure. One experimental theme common to most of these strategies is comparing the magnitude of the viral transcript and/or protein with and without activation of cells harboring the latent viruses. While technically simpler assays, such as DNA PCR, tend to overestimate the viral reservoir size, the contrary may be true for technically complicated assays. A range of experimental formats has been developed to estimate the size of the replication-competent viral reservoir; however, there is no single experimental format that satisfies all the requirements.

The principal approach for detecting and quantifying latently infected cells is the Quantitative Viral Outgrowth Assay (QVOA), which measures infectious units per a million of resting CD4^+^ T cells (11). Although QVOA is considered the golden standard for latent reservoir measurement, the application of this assay is limited due to the requirement for large quantities of blood and the complex experimental design. Several modifications to the original QVOA have been reported improving the assay sensitivity, dynamic range, and throughput (12–15). However, QVOA underestimates the latent reservoir as only a small proportion of replication-competent proviruses of infected cells produces virus following a single round of activation (6).

Alternative assays to measure LR size include PCR-based methods that quantify HIV DNA from enriched CD4^+^ cells using qPCR or droplet digital PCR (ddPCR). Despite their sensitivity and precision, PCR amplification-based assays fail to distinguish between intact and defective provirus and overestimate the LR size (15). Recently, Bruner et al. developed the intact proviral DNA assay (IPDA), which utilizes ddPCR to simultaneously detect two regions of the HIV-1 genome (Ψ sequence and *env*) often deleted in replication-deficient proviruses (16). Although IPDA is superior to the total HIV-1 DNA amplification, the assay still overestimates the proportion of the intact virus, as it overlooks defects in other regions of the viral genome (17, 18). Importantly, IPDA also is restricted by an additional technical limitation that the inducibility of intact proviral genomes identified by the assay remains to be demonstrated. Given this limitation, IPDA needs to be performed alongside inducible HIV RNA transcription assays to quantify the transcription-competent LR (16, 19, 20).

The splicing profile of the HIV-1 primary transcript is highly complex, regulated by four splice donors and eight splice acceptors and several additional cryptic, cis, and trans-regulatory elements (19, 21). Further, strain differences, subtype-specific variations, and host cell lineage and activation differences can add more complexity to the splicing of the viral transcript. Approximately 40-60 differently spliced transcripts classified into three broad categories may be identified intracellularly – un-spliced, partially spliced, and completely spliced transcripts. The completely spliced viral transcripts of approximately 1.8 kb code for the three early viral proteins - Tat, Rev and, Nef. Importantly, more than half of the early viral transcripts code for the Nef protein (21).

LR size may also be measured by quantifying the cell-associated HIV-1 RNA produced following *in vitro* stimulation (19, 20). Procopio et al. developed a new RNA-based method called the Tat/rev Induced Limiting Dilution Assay (TILDA), which measures the frequency of latently infected CD4^+^ T cells producing *tat/rev* multiply spliced (ms) HIV-1 RNA upon activation (22). The assay thus offers a relatively simple experimental format blended with a powerful detection strategy. Unlike DNA PCR, which amplifies the whole proviral DNA, TILDA identifies only the transcription-competent component of the latent reservoir, thus permitting a relatively representative estimate of the reservoir. Further, TILDA may also have a technical advantage over QVOA, which detects the actual infection units of a latent reservoir. This is because QVOA underestimates the size of replication-competent infectious units since only a minor proportion of the latent reservoir is activated to produce infectious viral particles following activation (6). Compared with DNA PCR and QVOA representing the two extremes of the detection strategies of HIV reservoir, TILDA occupies a central position on the detection spectrum (10, 23). Thus, although TILDA may overestimate the size of the replication-competent viral reservoir since all the cells producing viral transcripts may not produce infectious viral particles, the assay still offers technical simplicity and detection sensitivity.

TILDA, despite inherent limitations, represents a technically simple yet powerful strategy to evaluate HIV-1 latency reversal. However, the enormous magnitude of viral genetic diversity presents a technical challenge for a broad-level application of TILDA that warrants a solution, as correctly highlighted by Procopio et al. (24). TILDA, prioritizing the amplification of a smaller fragment for the real-time PCR, targets the viral regions that are not well conserved. Consequently, the amplification schema is skewed towards amplifying HIV-1B sequences and not even all the strains within this genetic subtype. Bertoldi et al. modified the TILDA for HIV-1C; however, the primers and probe designed are highly specific to HIV-1C and not likely to detect other HIV-1 genetic subtypes (25). The application of other TILDA formats adopted to match the genetic sequences of specific HIV-1 subtypes also is constrained by the limited detection breadth of diverse viral genetic families (26). Genetic variation among genetic subtypes of HIV-1 can span as high as 17-35% (27); hence a molecular assay is urgently needed to overcome the limitation of HIV-1 genetic diversity.

Here, we have enhanced the breadth of TILDA by targeting the regions of HIV-1 highly conserved among diverse viral genetic subtypes. The improvised assay, called universal TILDA (U-TILDA) for simplicity, can be instrumental in characterizing HIV-1 latent reservoirs.

## Materials and Methods

### Cell Culture

Jurkat cells (Clone E6-1) and J-Lat 8.4 cells were procured from ATCC and cultured in RPMI 1640 (catalog no. AL162S, HiMedia Laboratories, Mumbai, India), supplemented with 10% fetal bovine serum (catalog no. 10082147, Thermo Fisher Scientific, Waltham, USA), 100 units/ml penicillin G (catalog no. P3032, Sigma-Aldrich, Inc., St. Louis, USA), 2 mM L-glutamine (catalog no. G8540, Sigma-Aldrich, Inc., St. Louis, USA) and 100 μg/ml streptomycin (catalog no. S9137, Sigma-Aldrich, Inc., St. Louis, USA). Human Embryonic Kidney 293T (HEK293T) cells were cultured in Dulbecco’s Modified Eagle’s Medium (catalog no. D1152, Sigma-Aldrich, Inc., St. Louis, USA), supplemented with 10% FBS, 100 units/ml penicillin G, 2 mM L-glutamine, and 100 μg/ml streptomycin.

### Construction of Template Plasmid (pBSKS-MS1)

We constructed a bacterial plasmid containing a ~500 bp of spliced template sequence of HIV-1C to optimize the two primer pairs. The spliced viral RNA was generated in HEK293T cells transfected with a full-length Indie-C1 molecular clone. Briefly, HEK293T cells (0.5×10^6^ cells) were seeded in a 6-well plate one day before the transfection. The cells were transfected with the full-length pIndie-C1 plasmid using the calcium chloride transfection protocol. The medium was replenished six h post-transfection. Cells were harvested at 48 h post-transfection, and TRIzol Reagent (catalog no. T9424, Sigma-Aldrich, Inc., St. Louis, USA) was used to isolate total cellular RNA from the cells (28, 29). One μg of RNA was subjected to DNase treatment (catalog no. M0303S, New England Biolabs Inc., Ipswich, USA), to remove HIV-1 plasmid DNA. The RNA was then converted into the first-strand cDNA using an oligo-dT primer and a Tetro cDNA synthesis kit (catalog no. BIO-65043, Bioline, London, UK) in 20 μL reaction volume. The reaction vials were incubated at 65°C for 5 min, following 2 min incubation on ice and 50°C for 30 min. The reactions were terminated by incubating the samples at 85°C for 5 min followed by RNaseH treatment. One μL of the cDNA was then subjected to PCR amplification using the outer round primers (N2830 and N2831). The amplified product was cloned in pBluescript II KS digested with EcoRV. The recombinant clones were identified using a diagnostic PCR and RE analysis using EcoRI and SalI. One of the recombinant plasmids labeled as pBSKS-MS1 was used as a template to optimize the PCR conditions.

### Optimization of the Nested Real-Time PCR Using a Plasmid Template

A real-time PCR format was optimized for the external (N2830 and N2831) and internal primer (N2832 and N2833) pairs independently (see **Table 1** for primer sequences). Parameters, such as the annealing temperature, extension time, number of cycles required, were optimized to values that gave optimal results. Once the amplification conditions of the two primer pairs were optimized, we integrated the two PCRs to develop a nested PCR. The template plasmid (pBSKS-MS1) was serially diluted (10^4^ to 10^0^) and used as a template for the first round. The first round was performed using a conventional thermocycler (Peqlab Biotechnologie GmbH, Erlangen, Germany) in 25 μL PCR mix containing 5 μL of template, 2.5 μL of 10X standard *Taq* reaction buffer, 500 nM of each primer (N2830 and N2831), 200 mM dNTPs, and 1 U of *Taq* polymerase (M0273S, New England Biolabs Inc., Ipswich, USA). The cycling conditions were initial denaturation at 95°C for 3 min, followed by 25 cycles of 95°C for 15 s, 60°C for 30 s, and 72°C for 30 s. The first-round PCR product was used as a template for the second-round, nested, real-time PCR amplification, performed using a CFX96 Touch Real-Time PCR Detection System (Bio-Rad Laboratories, California, USA). The 25 μL PCR mix containing 1 μL of the template (first-round product), 5 μL of 5X MyTaq Reaction Buffer, 500 nM of each primer (N2832 and N2833), 200 nM of the TaqMan probe (N2492), and 1.25 U of My*Taq*™ DNA polymerase (catalog no. BIO-21105, Bioline, London, UK) was used for the second-round amplification. The cycling conditions were initial denaturation at 95°C for 3 min, followed by 40 cycles of 95°C for 15 s and 60°C for 1 min.

**Table 1:** Sequences of primers and probe used for PCR and cDNA synthesis.

Oligonucleotides	Identity	Sequence (5’-3’)
Primers	N2830 (OFP)	CTGCTTAAGCCTCAATAAAGCTTGCCT
	N2831 (ORP)	CCTGTGCCTCTTCAGCTACCACCGATTGAG
	N2832 (IFP)	GACTCTGGTAACTAGAGATCCCTCAGA
	N2833 (IRP)	GAATCGAAGAAGAAGGTGGAGAGCAAGACA
Probe	N2842	(FAM)-CTCTCGACGCAGGACTCGGCTTGCTGA-(BHQ-1)
RT primers	N4101	TTTTTTTTTTTTTTCAGAGCACTC
	N4102	TTTTTTTTTTTTTCAGAGCACTCAAG
	N4103	TTTTTTTTTTTTTTCAGAGCACTCAAGG
Genomic DNA primers/Probe	N2208	GATCTGAGCCTGGGAGCTCTCTG
	N2209	GACTCTGGTAACTAGAGATCCCTCAGA
	N2210	(FAM)-CTGCTTAAGCCTCAATAAAGCTTGCCTTGAGTGCT-(TAMRA)

OFP, Outer forward primer; ORP, Outer reverse primer; IFP, Inner forward primer; IRP, Inner reverse primer. Reverse primer sequences are presented as reverse complement.

### Optimization of the Nested Real-Time PCR Using cDNA Template

One million Jurkat cells in a six-well plate were pulsed with a defined and varying number of J-Lat 8.4 cells (0 to 10,000 cells, in a ten-fold serial dilution), containing a single copy of a provirus. The cells were activated with a cocktail of activators comprising 5 ng/ml PMA (catalog no. P1585, Sigma-Aldrich, Inc., St. Louis, USA), 10 ng/ml TNFα (catalog no. 130-094-019, Miltenyi Biotech, Bergisch Gladbach, Germany), and 2.5 mM HMBA (catalog no. 224235, Sigma-Aldrich, Inc., St. Louis, USA) for 24 h or left untreated. Total cellular RNA was extracted from triplicate wells of activated and control wells using TRIzol Reagent (catalog no. T9424, Sigma-Aldrich, Inc., St. Louis, USA). Five hundred ng of RNA was subjected to DNase treatment (catalog no. M0303S, New England Biolabs Inc., Ipswich, USA), to remove DNA contamination. The total RNA was reverse transcribed in a 20 μL reaction volume to generate cDNA using an oligo-dT primer or a pool of three HIV-specific oligo-dT primers (**Table 1**) and a commercial kit (Tetro cDNA synthesis kit, catalog no. BIO-65043, Bioline, London, UK). A nested real-time PCR was performed using the cDNA as described above. In parallel, GFP expression was monitored from cells with and without activation using a flow cytometer (BD FACS Aria III 1018 sorter, BD biosciences, New Jersey, USA).

### Droplet Digital PCR

cDNA was prepared as described above using the total cellular RNA extracted from HEK293T cells transfected with the pIndie-C1 molecular clone. One or 0.5 μL of the cDNA template was used as the template for ddPCR. The template DNA, plasmid template, or cDNA, was used in the assay at two different concentrations. The ddPCR reaction mix consisted of 10 µl 2x ddPCR super mix for probes (Bio-Rad Laboratories, California, USA); 900 nM primers (N2832 and N2833); 250 nM probe (N2492), and 1 µl of the template DNA in a final volume of 20 µl. The total reaction mix was placed in an 8-channel cartridge, and 70 µl of droplet generating oil was added. The samples were loaded to the QX100 droplet generator (Bio-Rad Laboratories, California, USA), and droplets were formed, following the manufacturer’s instructions. The contents were transferred to a 96-well plate and placed on a T100 Thermal Cycler (Bio-Rad Laboratories, California, USA) after sealing. The PCR conditions were as follows: 95°C for 5 min, followed by 40 cycles of 94°C for 15 secs, 60°C for 1 min, and a final 10 minutes at 98°C for enzyme inactivation. The droplets were subsequently read automatically by the QX100 droplet reader (Bio-Rad Laboratories, California, USA Laboratories). The data were analyzed with the QuantaSoft analysis software 1.3.2.0 (Bio-Rad Laboratories, California, USA). We also performed ddPCR with a two-fold dilution series (4,000, 2,000, 1,000, 500, and 250 copies) of plasmid DNA and a three-fold dilution series (1 µL neat, 3, 9, 27, and 81-fold dilutions) of cDNA template using N2832, N2833, and N2842 (probe).

### cDNA Generation From Different HIV-1 Subtype Molecular Clones

HIV-1 molecular clones representing four diverse viral genetic subtypes (A, B, C, and D) were procured from the AIDS Reagent Program at the National Institutes of Health. The seven infectious viral molecular clones included - Q23-17 (Cat No.12649, A), pTRJO.c/2851 (Cat No.11747, B), LAI (Cat No.2532, B), NL4-3 (Cat No. 114, B), Z3576M (Cat No.13259, C), and 94UG114.1.6 (Cat No.4002, D). Indie C1 molecular clone of HIV-1C (Genbank accession number AB023804) was a kind gift of Dr. Masashi Tatsumi, Department of Pathology, International Medical Center of Japan, Tokyo. The infectious molecular clone UC57703357c02 (HIV-1E) was a kind gift from Dr. Sodsai Tovanabutra, The Henry M. Jackson Foundation for the Advancement of Military Medicine, Inc., the U. S. A. HEK293T cells at 40% cell confluence, seeded in a 6-well culture dish, were transfected independently with 4 μg of individual plasmid clones. pCAG-tdTomato (30 ng) was used as an internal transfection control. The medium was replenished 6 h post-transfection. The total cellular RNA was extracted, cDNA synthesized, and the nested real-time PCR was performed as described above. Glyceraldehyde-3-phosphate dehydrogenase (GAPDH) reference gene PCR was used for the normalization of the real-time PCR data.

### Study Participants

Participants for this study were recruited at the Indira Gandhi Institute of Child Health, Bangalore. The study participants were ART naïve or have been on ART for a year. From medical records, the participants belong to the chronic phase of the viral infection although the date of infection is not known. Depending on the molecular typing of the LTR, all the study participants of the present study were infected with HIV-1 subtype C viral strains (Data not shown). Written informed consent was obtained from all study participants following the approval of the institutional review board. The Human Ethics and Biosafety Committee of Jawaharlal Nehru Centre for Advanced Scientific Research (JNCASR), Bangalore, reviewed the proposal and approved the study.

### TILDA Using Primary CD4^+^ T Cells

CD4^+^ cells were enriched from stored PBMCs using a negative magnetic selection kit (EasySep Human CD4^+^ T cell Isolation Kit, catalog no. #19052, Stemcell Technologies Inc., Vancouver, Canada). The purity of enriched CD4^+^ T cells was validated by flow cytometry after staining with PE Mouse Anti-Human CD4^+^ antibody (catalog no. 561844, BD biosciences, New Jersey, U.S.) and PE Mouse IgG1, κ Isotype Control (catalog no. 555749, BD biosciences, New Jersey, U.S.).Enriched CD4^+^ T cells were resuspended at 2 × 10^6^ cells/ml in RPMI 1640 supplemented with 10% fetal bovine serum, 100 units/ml penicillin G, 2 mM L-glutamine, and 100 μg/ml streptomycin and rested for 3–5 h at 37°C. The cells were activated for 12 h using 100 ng/ml PMA (catalog no. P1585, Sigma-Aldrich, Inc., St. Louis, USA Aldrich) and 1 μg/ml Ionomycin (catalog no. I0634, Sigma-Aldrich, Inc., St. Louis, USA Aldrich). After 12 hr of activation, cells were counted and serially diluted in cell culture medium to 27x10^6^ cells/ml, 9x10^6^ cells/ml, and 3x10^6^ cells/ml. One μL of the cell suspension from each stock corresponding to 27 000, 9 000, and 3 000 cells, respectively, was added to 16 replicate wells containing 5 μL of 2X reaction buffer, 0.1 μL of RNase inhibitor, 0.2 μL of 25 μM each primer (N2830 and N2831), 0.5 μL of 2 μM HIV-specific cDNA primer pool, 2.8 μL of H_2_O, and 0.2 μL of Superscript III Platinum *Taq* (SuperScript III Platinum One-Step qRT-PCR Kit, catalog no. 11732020 Invitrogen, Carlsbad, California). The amplification was carried out at 50°C for 15 min, denaturation at 95°C for 2 min, 25 cycles of amplification (95°C for15 s, and 60°C for 4 min). At the end of the PCR, the amplified product was diluted using 40 μL of TE, and 1 μL of this sample was used as a template for the second round of PCR using My*Taq*™ DNA polymerase (catalog no. BIO-21105, Bioline, London, UK). This reaction was performed by adding 2 μL of the 5x My*Taq* Reaction Buffer, 2 μL of each of the primers (N2832 and N2833, both at 10 μM), 0.25 μL of the HIV probe at 5 μM, and 3.4 μL H_2_O to each well, in a 10 μL final reaction volume. The real-time PCR reaction was performed using the following program, Pre-incubation at 95°C for 10 min, 40 cycles of 95°C 10 s, 60°C 30 s, and 72°C 30 s. Positive wells were counted, and the maximum likelihood method was used to calculate the frequency of cells with inducible HIV msRNA (http://bioinf.wehi.edu.au/software/elda).

### The Estimation of Total HIV-1 DNA and p24

We extracted genomic DNA from one million CD4^+^ cells using a commercial DNA extraction kit (GenElute Blood Genomic DNA kit, Cat. No. NA2020, Sigma Aldrich, U.S.A.). The genomic DNA was eluted in 200 μL of the elution buffer. A sequence of 130 bp in the R-U5 region was amplified using 5 µL of genomic DNA as the template. The primer pair N2208-N2209 in combination with the probe N2210 FAM was used for the ddPCR (see **Table 1** for oligo sequences). The levels of p24 secreted into the culture medium by one million CD4^+^ cells, activated with 100 ng/ml of PMA and 1 μg/ml Ionomycin, were determined 72 hours following the cell activation, using a commercial kit (Catalog no. IR232096, J.Mitra & Co. Pvt. Ltd., New Delhi, India).

### Statistical Analysis

The data were analyzed using GraphPad Prism 5, and P values of 0.05 or less were considered statistically significant. Baseline and induced TILDA values were compared using the Wilcoxon matched-pairs signed-rank test. Spearman’s rank correlation analysis was used to correlate TILDA values with HIV-DNA and p24. Paired *t*-test was used to evaluate the statistical significance of the reverse transcription primers (oligo dT *vs.* HIV-specific oligo-dT), and B-TILDA *vs.* U-TILDA.

## Results

### The Primers and the Probe Target The Most Conserved Sequences in the Viral Genome

**Figure 1:**
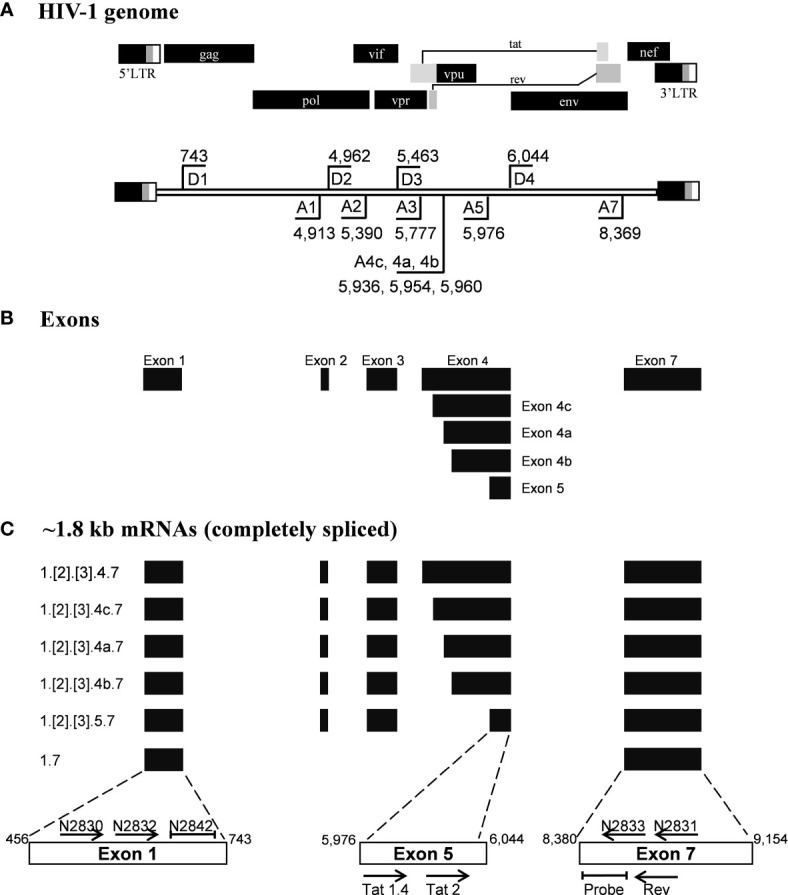
Schematic representation of the HIV-1 exon profile, early transcripts, and primers. **(A)** A schematic presentation of HIV-1 genome (Upper panel). The locations of splice donor (D1-D4) and splice acceptor sites (A1-A5, A7) are depicted on the HIV-1 genome flanked by the LTRs (Lower panel). The schematic is not drawn to the scale. The coordinates presented are as per the HIV-1 genome of the HXB2 reference molecular clone. **(B)** The major exons of HIV-1 are represented by solid horizontal bars. **(C)** The profile of the early viral transcripts: Several alternatively spliced viral transcripts are formed within the 1.8 kb group. Numbers at the left margin depict the exons that are spliced together to form a specific viral transcript. The exons shown in square brackets are included differentially in the transcript formation. The horizontal arrows depict the primers used in the assays and their orientation. The primer positioning is not to the scale. The primers and probes shown above the open boxes are used in the present work, and those shown below the boxes were reported previously by Procopio et al. Note that the non-coding exon-1 and the 3’-end exon-7 are present in all the viral transcripts, regardless of the splicing differences.

**Figure 2:**
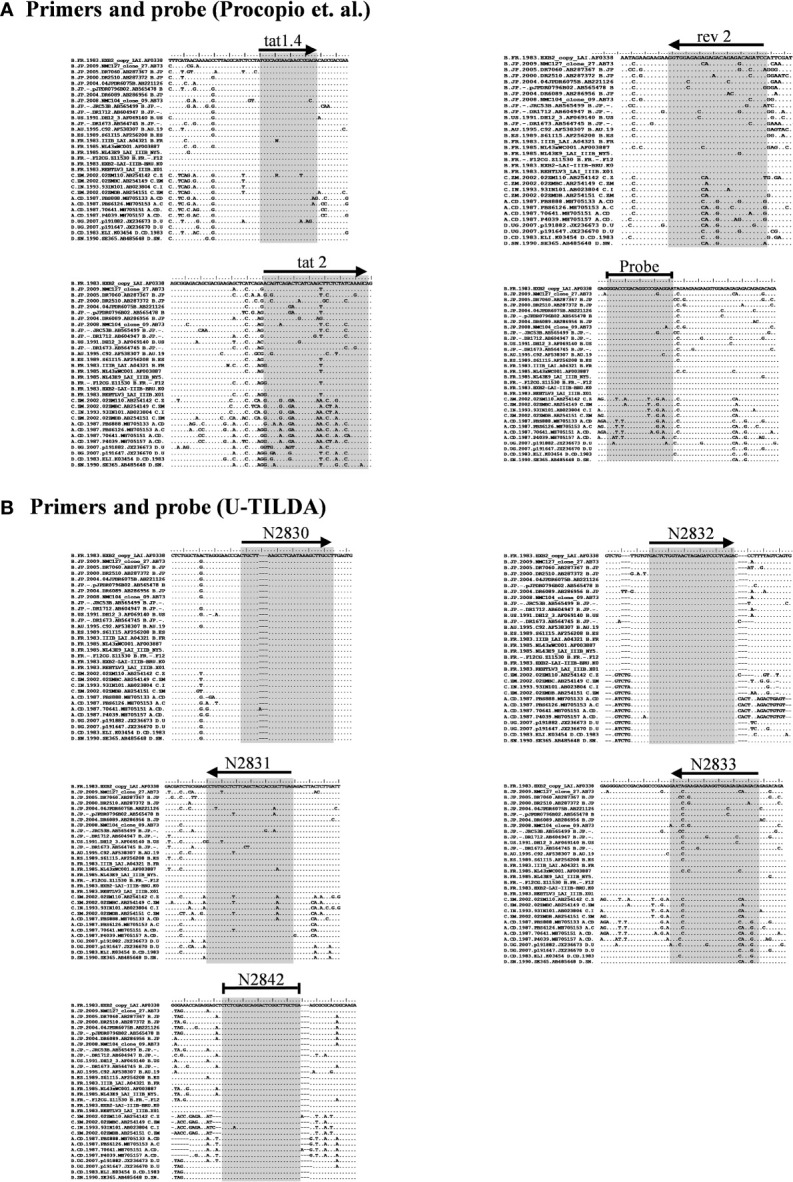
Multiple sequence alignment of representative viral strains. **(A)** The primers and probe of Procopio et al. **(B)** The primers and probe of the U-TILDA. The regions targeted by the primers and probes of several strains belonging to the major HIV-1 subtypes were downloaded from the Los Alamos HIV Sequence Database. The alignment of a few representative sequences of each subtype is presented here. The target sequences are highlighted by shading. The horizontal arrows represent the orientation of the primers. A blunted horizontal line represents probes. Dots represent sequence identity and dashes deletions. The labels of the oligos represent their identity.

Given the merits of TILDA, the primary aim of the present work is to enhance the breadth of detection of this assay format by accounting for high magnitude genetic variation of HIV-1 subtypes. The sensitivity and breadth of a PCR amplification depend on the magnitude of sequence conservation that the primers target in the viral genome when the levels of the viral transcripts expressed by cells remain a constant factor. The reported primers of TILDA target exons-5 and -7 of HIV-1 taking advantage of the presence of a ~2,325 bp intron sequence between the exons (**Figures 1A, B**). The strategy of primer placement is expected to permit the amplification of the 1.8 kb early viral transcripts. While the strategy efficiently discriminates the early viral transcripts from partially spliced and un-spliced viral transcripts and the proviral genomic DNA, the regions selected for primer are not well conserved, thus, compromising the efficiency and breadth of TILDA (**Figure 2A**). Thus, the combination of these primers and the probe cannot amplify the diverse viral strains of HIV-1 characterized by a high genetic variation.

We have given the highest priority to sequence conservation while designing the primers and probe for TILDA. To this end, we elected to target exon-1 and exon-7 for positioning the primer and probe sequences. Of note, these two exons are invariably present in all viral transcripts regardless of alternative splicing (**Figure 1**). The two forward primers (N2830 and N 2832, see **Table 1** for oligo sequences) and the probe (N2842) are positioned on exon-1 (**Figure 1C**), which is one of the highest conserved regions in the viral genome (**Figure 2B**). Likewise, we chose two regions in exon-7 highly conserved among the diverse HIV-1 subtypes to position the two reverse primers (**Figure 2B**). Despite sequence conservation, the new primer and probe combination is expected to amplify relatively larger-sized fragments in the PCR. Since the primers are positioned in exon-1 and -7, flanking the differentially spliced exons 2, 3, 4A, 4B, 4C, and/or 5, the PCR products generated in our assay, which we refer to as universal TILDA (U-TILDA), for simplicity, represent a pool of several early viral transcripts (**Table 2**). The PCR products of the first round are expected to range between 390-782 bp and those of the second round between 236-628 bp. Thus, the primer design in our assay format circumvents the problem of HIV-1 genetic diversity and amplifies a pooled species of PCR products representing the early viral transcripts. Although the primers and the probe can also anneal to the cDNAs of un-spliced and partially spliced viral transcripts, these fragments are refractory to amplification given the larger size.

**Table 2:** The expected size of various HIV-1 early viral transcripts in the TILDA.

mRNA	Transcript	Exon combination	The expected size of the PCR fragments (bp)
**Tat**	Tat 1	1.4.7	(167 + 268+69) 504
	Tat 2	1.[2].4.7	(167 + 50+268+69) 554
	Tat 3	1.[3].4.7	(167 + 74+268+69) 578
	Tat 4	1.[2].[3].4.7	(167 + 50+74+268+69) 628
**Nef**	Nef 1	1.7	(167 + 69) 236
	Nef 2	1.5.7	(167 + 69+69) 305
	Nef 3	1.[2].5.7	(167 + 50+69+69) 355
	Nef 4	1.[3].5.7	(167 + 74+69+69) 379
	Nef 5	1.[2].[3].5.7	(167 + 50+74+69+69) 429
**Rev**	Rev 1	1.4B.7	(167 + 85+69) 321
	Rev 2	1.4A.7	(167 + 91+69) 327
	Rev 3	1.4C.7	(167 + 109+69) 345
	Rev 4	1.[2].4B.7	(167 + 50+85+69) 371
	Rev 5	1.[2].4A.7	(167 + 50+91+69) 377
	Rev 6	1.[2].4C.7	(167 + 50+109+69) 395
	Rev 7	1.[3].4B.7	(167 + 74+85+69) 395
	Rev 8	1.[3].4A.7	(167 + 74+91+69) 401
	Rev 9	1.[3].4C.7	(167 + 74+109+69) 419
	Rev 10	1.[2].[3].4B.7	(167 + 50+74+85+69) 445
	Rev 11	1.[2].[3].4A.7	(167 + 50+74+91+69) 451
	Rev 12	1.[2].[3].4C.7	(167 + 50+74+109+69) 469

The exons in square brackets are the alternatively-spliced transcripts.

### High-Sensitive Detection of an HIV-1 Template

**Figure 3:**
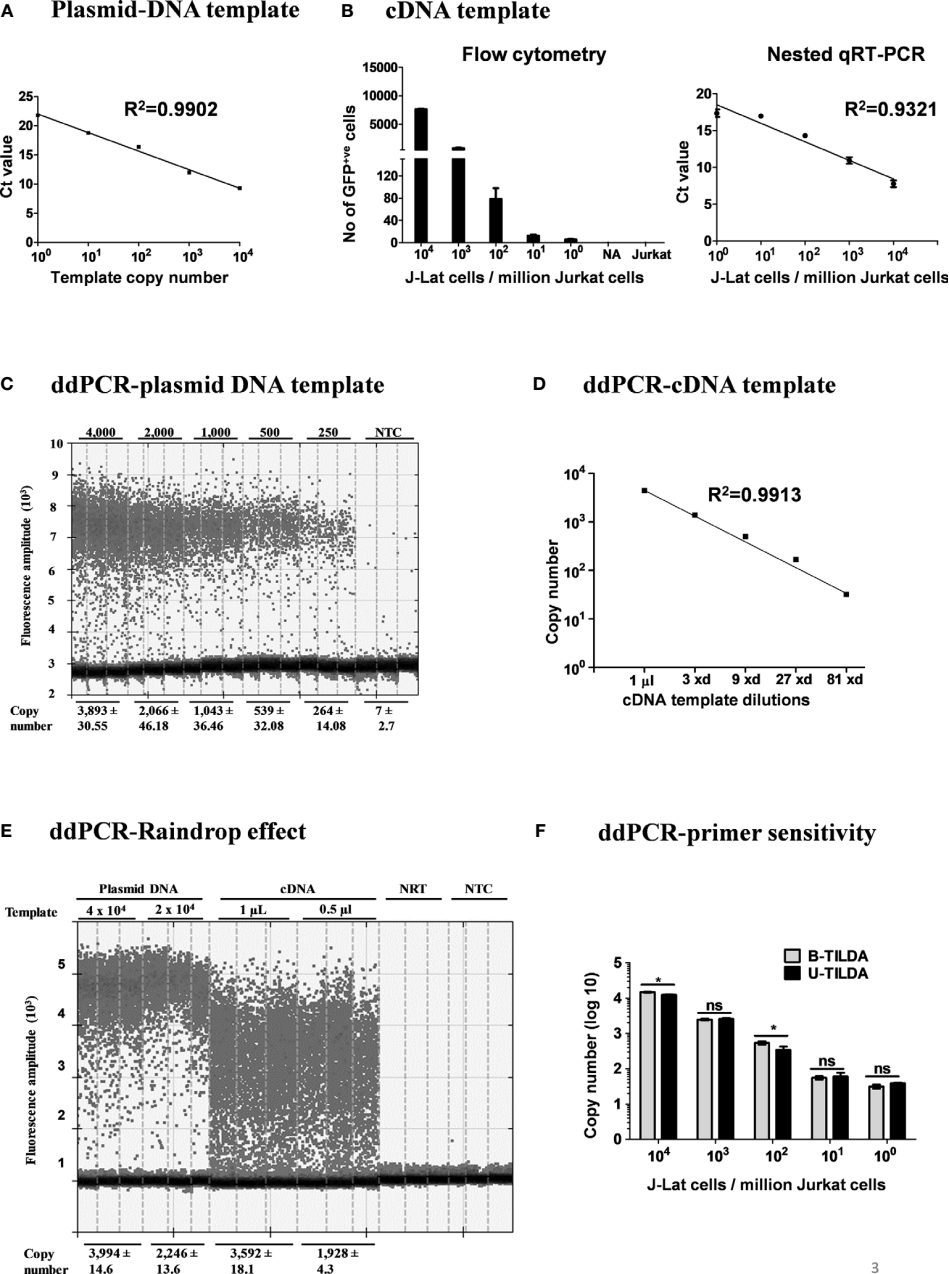
Amplification efficiency of the new primer-probe set. **(A)** Standard curve of amplification using a plasmid DNA, pBSKS-MS1, at a defined copy number (10^4^-10^0^ copies). The data are representative of three independent experiments performed in triplicate. Each data point on the graph represents the Mean ± SD of three Ct replicate values. **(B)** Flow cytometry analysis of J-Lat 8.4 cells pulsed into a background of 10^6^ Jurkat cells and then activated (left panel). cDNA of the viral transcripts of the activated cells was used as the template for amplification. The right panel represents the standard curve of the C_t_ values (Mean ± SD) of three independent assay done in triplicates with inter-assay CV of 5%. **(C)** ddPCR of serially diluted pBSKS-MS1 plasmid using U-TILDA primers and probe. **(D)** ddPCR of serially diluted J-Lat cDNA sample prepared as in panel **(B)**. **(E)** ddPCR performed using cDNA of the RNA extracted from HEK293T cells transfected with pIndie C1 molecular clone or pBSKS-MS1 as the template. The template copy number is indicated at the top of the lanes. Each assay contained three replicate wells, and each lane represents one well. The dark dots at the bottom of each lane represent the negative droplets with no fluorescence (no amplification), and the grey dots above represent the positive droplets. The number of accepted droplets for each well ranges from 14,000-17,000. Copy number was determined using Poisson’s calculation. The number of positive droplets is indicated at the bottom of the wells. The data are representative of two independent experiments (Left panel). A standard curve of amplification is presented (Right panel). NRT, No reverse transcriptase control; NTC, No template control. **(F)** The primer-probe sets of B- and U-TILDA formats have been compared in a ddPCR using defined numbers of J-Lat cells serially diluted into one million Jurkat cells before cell activation as in panel **(B)** A Paired *t*-test was performed to compare the data. (*p < 0.05, ns, not significant).

After optimizing the various experimental parameters, we evaluated the sensitivity of the nested-PCR using a plasmid DNA template pBSKS-MS1, which harbors a target sequence of approximately 500 bp representing one specific early viral transcript of HIV-1C. The plasmid DNA was diluted serially using a solution supplemented with salmon-sperm genomic DNA to provide the complex genetic background during amplification. The amplification of the first round was performed for 25 cycles. One µl of the PCR reaction mix was transferred to the second round of the amplification of 35 cycles. The second round of amplification was monitored using a TaqMan probe. The real-time PCR amplified the target sequence with high sensitivity (**Figure 3A**) and detected two or three copies of the template DNA, in a highly reproducible manner. However, a plasmid DNA template may not represent the enormous genomic background complexity of heterogeneous viral and cellular mRNA.

To this end, we evaluated the assay sensitivity to detect early viral transcripts induced in J-Lat T cells after cellular activation. J-Lat cells (Clone 8.4) harbor a full-length HIV-1B genome that contains a defective envelope and encodes GFP substituted for Nef. J-Lat cells represent an excellent cell model for HIV-1 latency as the production of the viral transcripts is activated many folds following cell activation. We pulsed one million Jurkat T cells with a range of J-Lat cells (10^4^, 10^3^, 10^2^, 10^1^, 5, and 0 cells). The cell pool was then activated with a cocktail of activators (PMA, TNFα, and HMBA) for 24 h or left untreated. Flow cytometry of cells confirms the activation of the pulsed J-Lat cells as these cells upregulate the expression of virus-encoded GFP (**Figure 3B**, left panel). Total cellular RNA was extracted, cDNA was synthesized using oligo dT, and one µL of the cDNA reaction mixture was used in the real-time PCR, as described above. An excellent inverse correlation was identified between the Ct value of the PCR and the number of the J-Lat cells pulsed and activated (**Figure 3B**, right panel). There was no amplification when cells were not activated. Using the optimized conditions, we could detect cDNA from as few as five activated proviruses with high reproducibility.

Importantly, our assay is expected to amplify not a single product of a defined size but a pool of various early viral transcripts (**Table 2**). A variation in the size of the amplified products may lead to a concomitant variation in the overall amplification efficiency. The possibility of compromised amplification sensitivity, however, was ruled out from the above experimental data (**Figure 3B**) as the assay can detect very low copy numbers of the early viral transcripts. Nevertheless, to examine if the size difference of the amplified products can affect the amplification efficiency of individual viral early transcripts, we applied droplet digital PCR (ddPCR). Before using viral cDNA in the ddPCR, we confirmed a positive correlation between the input copy number and the number of fluorescent droplets using pBSKS-MS1plasmid described above as the template. A perfect correlation was evident between the template plasmid copy number used in the PCR, ranging from 4,000 to 250, and the droplets observed following the PCR (**Figure 3C**). Additionally, a perfect correlation was also observed when the cDNA of HIV-1C Indie-C1 viral transcripts produced in HEK293T cells was serially diluted in a three-fold dilution series and used in the ddPCR (**Figure 3D**).

Although different early viral transcripts bind the same primer pair since the target sites are present on all the viral transcripts, the intervening sequences between the primer-binding sites are expected to vary depending on which internal exons are included in each different transcript due to alternative splicing (**Figure 1C** and **Table 2**). At the end of the second round of PCR, at least 21 differently sized PCR products ranging from 236 bp to 628 bp are expected to be present in the reaction mix. Typically, the overall amplification efficiency of the PCR is inversely correlated to the size of the amplified product. Thus, a shorter viral transcript may amplify with greater efficiency than a longer one. The fluorescence intensity of individual droplets in ddPCR may vary depending on the amplification efficiency in a single droplet. If this expectation holds, the droplets may manifest a phenomenon of ‘the raindrop effect’ where the droplets will spread across the Y-axis above a defined amplification threshold line.

To test for this possibility, we used the viral cDNA of Indie-C1 produced as described above in ddPCR at two different concentrations (**Figure 3E**). As an additional control, we used pBSKS-MS1 plasmid DNA described above. In the lanes committed to the plasmid DNA template, a clear demarcation was evident between the positive and negative droplets with the average fluorescence intensity difference of four to five folds (**Figure 3E**). Further, approximately 2,000-4,000 of the 17,000 total droplets were positive at the higher concentration of the plasmid DNA template. Thus, the ddPCR represented a non-saturating experimental condition where the positive droplets were not expected to contain more than a single template copy per droplet.

In contrast, the positive droplets of the cDNA template exhibited a full-range variation of fluorescence intensity. Despite the spread of the positive droplets, the droplets of low fluorescence intensity can be demarcated from the negative droplets. A good correlation was evident between the number of positive droplets and the input template copies. Additionally, 10-20% of the droplets contained the template DNA (approximately 2,000-4,000/17,000). A good agreement is evident among the replica wells of the assay. The two negative controls of the assay did not show a positive amplification. Importantly, the genetic diversity of the virus could also contribute partially to the ‘raindrop effect’, especially if the source of the cDNA is a primary viral strain. However, the effect of genetic diversity in this specific experiment was expected to be minimal as we used a molecular clone as the template. Thus, the amplification heterogeneity seen in the experiment must be due primarily to viral transcript length variation.

Further, we compared the amplification efficiency of the conventional TILDA, referred to as B-TILDA here for simplicity, with that of U-TILDA using viral cDNA sourced from J-Lat cells as described above in panel B. A defined number of J-Lat cells (10^4^, 10^3^, 10^2^, 10^1^, and 10^0^ cells) were pulsed into one million Jurkat cells before activation. Total RNA was extracted from the cells and converted into cDNA as described and used to compare the two assay formats. The primer and probe sequences of both the assays match well with the target viral sequences despite a few variations of less significance. Both the TILDA formats detected the target sequence with comparable efficiency, demonstrating good dose-response (**Figure 3F**). In summary, despite the amplification heterogeneity, U-TILDA performed robustly without a loss in the overall amplification sensitivity.

### HIV-1 Genetic Diversity Does Not Compromise U-TILDA Efficiency

**Figure 4:**
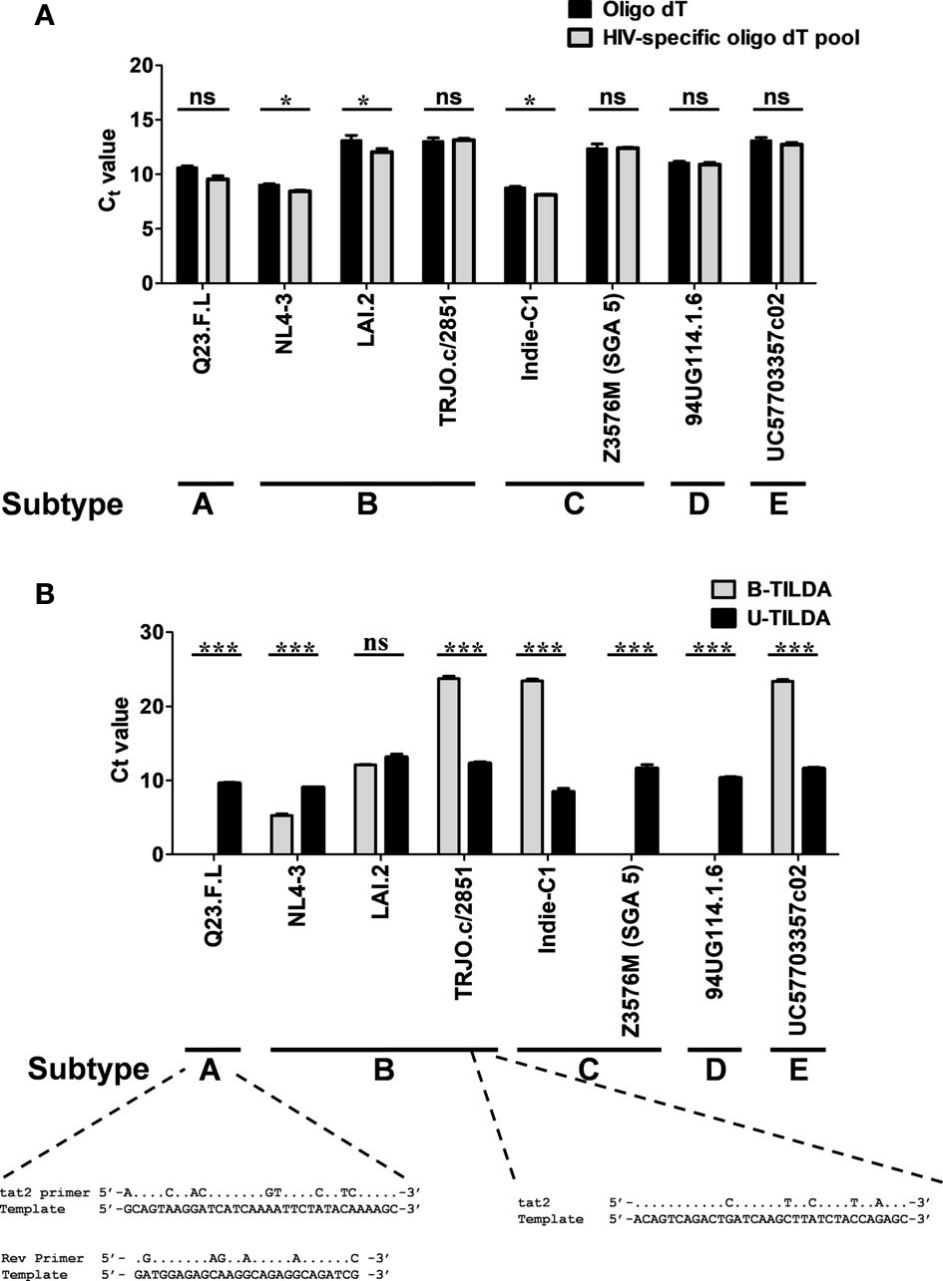
The assay detects the diverse genetic subtypes of HIV-1 with comparable efficiency. **(A)** The assay was performed using cDNA of the total RNA extracted from HEK293T cells transfected with one of the eight DNA plasmid vectors representing five major HIV-1 genetic subtypes, as shown. The reverse transcription was performed using the generic oligo-dT (filled bars) or a pool of three HIV-1-specific oligo-dT primers (grey bars). Data are representative of two independent experiments performed in triplicates. The data are normalized against GAPDH transcripts and presented as Ct value Mean ± SD. Paired *t*-test was used for the statistical evaluation (*p<0.05, ***p<0.001, and ns – not significant). **(B)** B-TILDA is severely impacted by HIV-1 genetic diversity. Viral cDNA samples prepared as in panel A above were used to compare the performance of B-TILDA (grey bars) Vs. U-TILDA (filled bars) formats. The data are normalized against GAPDH transcripts and presented as Ct value Mean ± SD. The sequence diversity of two viral strains with the B-TILDA primers has been presented where the assay failed (Q23.F.L) or the amplification efficiency was profoundly compromised (TRJO.c/2851). The dots represent sequence identity.

To examine if the optimized U-TILDA PCR can identify different viral strains regardless of genetic diversity, we used eight infectious molecular clones of HIV-1 belonging to five major genetic subtypes (A, B, C, D, and E). HEK293T cells were transfected with the individual viral plasmid vectors, and the total cellular RNA was isolated. A defined quantity of the cellular RNA was reverse transcribed using an oligo-dT primer or a pool of three HIV-1-specific oligo-dT primers (**Table 1**). Unlike the oligo-dT primer, which can anneal to all the cellular transcripts containing a poly-A tail, HIV-1-specific oligo-dT primers preferentially anneal the viral transcripts. One µl of the RT-PCR product was used as the template for the optimized nested-PCR, as described above. It is evident from the results that the optimized assay can detect all the eight viral clones belonging to five major HIV-1 genetic subtypes with comparable efficiency (**Figure 4A**). The minor differences noted in the Ct values may be ascribed to experimental variation rather than a genetic variation of the target sequences. We have ensured the quality and quantity of the viral plasmid vectors used in the assay and the transfection efficiency of HEK293T cells. The results were normalized against an internal control targeting GAPDH, which functioned with a uniform amplification efficiency of a Ct value of 20.98 to 21.75. The pool of HIV-1-specific oligo-dT primers worked with comparable or even marginally superior efficiency than the generic oligo-dT primer, although this improvement was not of great significance.

We next compared the B-TILDA with our assay to understand how genetic variation may impact the performance of the two assays. We used cDNAs generated from the eight HIV-1 infectious molecular clones representing five major HIV-1 subtypes (**Figure 4B**). As is evident, U-TILDA detected the transcripts of all the eight viral molecular clones and with comparable efficiency regardless of genetic diversity (Black bars). In contrast, B-TILDA detected only five of the eight molecular clones and failed to detect three clones completely, belonging to non-B subtypes – A-Q23.F.L, C-Z3576M (SGA 5), and D-94UG, representing subtypes A, C, and D, respectively. Further, B-TILDA required more amplification cycles for the positive identification of three of the five molecular clones (TRJO.c/2851, Indie-C1, and UC57703357c02 representing subtypes B, C, and E, respectively) as compared to U-TILDA. For example, while U-TILDA required a Ct value of only 12.4 cycles for the detection of the TRJO.c/2851 viral strain, B-TILDA needed a Ct value of 23.8, an additional 11.4 cycles, for the detection of this strain. Sequence comparison of the primers shows that the ‘tat2’ primer used in B-TILDA contains mismatches at five different positions with the target sequence of the TRJO.c/2851 viral strain, two of the mismatches located close to the 3’end of the primer, that severely reduced the amplification efficiency of the PCR (**Figure 4B**). Likewise, several sequence mismatches spanning the length of two primers (tat2 and Rev) of B-TILDA and the target sequence abrogated the amplification of Q23.F.L (**Figure 4B**) and C-Z3576M (SGA 5), and D-94UG viral strains (not shown). Thus, sequence mismatches compromised the amplification efficiency of B-TILDA significantly (TRJO.c/2851, Indie-C1, and UC57703357c02) or completely (Q23.F.L, C-Z3576M (SGA 5), and D-94UG) in detecting the HIV-1 viral strains. In contrast, U-TILDA amplification was not affected by the genetic variation of the eight HIV-1 viral strains examined, as the primers and the probe target highly conserved viral sequences. The data collectively ascertain that U-TILDA is robust enough to examine HIV-1 latency reversal kinetics given the broader range of detection and high sensitivity.

### U-TILDA Can Measure the Frequency of Persistently Infected Cells in Blood Samples

We evaluated the production of HIV-1 early transcripts from stored PBMC of 12 study participants with or without cell activation. The details of the participants have been summarized (**Table 3**). While six of these participants were ART-naïve, six other participants have been on ART for approximately a year at the time of blood collection. The mean CD4^+^ cell counts of the ART-naïve and ART-exposed groups were 210.6 ± 73.1 and 540.1 ± 168.4 cells/µl, respectively. The mean plasma viral load of the ART-naïve participants was 11,500 ± 15,901 copies/ml, whereas no plasma viral load was detected in the ART-exposed group. The mean CD8 cell counts of the ART-naïve and ART-exposed groups were 991.7 ± 194.4 and 1,352 ± 309.7 cells/µl, respectively.

**Table 3:** Participant details.

Participant Id	Gender		Age (years)		Duration of ART (in years)	CD4^+^ cell count (cells/µl)	Nadir CD4^+^ cell count (cells/µl)	CD8 cell count (cells/µl)	PVL (copies/ml)	Months on ART with PVL lower than DL	Cells expressing tat rev msRNA/10^6^ CD4^+^ cells	Confidence interval (95%)
S215_02 ( 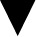 )	M		24		0	140.4	140.4	1,083.8	22,000	–	344	234-560
S326_02 ( 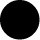 )	M		43		0	169.2	169.2	1,188.5	35,000	–	128	83-197
S338_02 ( 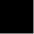 )	M		45		0	329.0	295.3	1,055.9	23,000	–	705	390-1,175
S119_02 ( 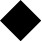 )	F		31		0	203.8	203.8	625.2	13,000	–	111	78-178
S137_02 ( 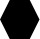 )	F		34		0	265.4	265.4	958.1	190,000	–	63	51-87
S187_02 ( 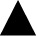 )	M		41		0	155.7	155.7	1,038.5	410,000	–	200	142-278
S398_06 ( 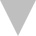 )	M		40		1	311.3	204.8	1,213.9	<DL	4	244	153-387
S073_06 ( 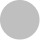 )	M		38		1	645.5	167.0	1,885.6	<DL	6	77	50-118
S371_06 ( 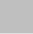 )	F		41		1	484.7	248.0	1,014.3	<DL	6	630	357-1,011
S137_06 ( 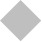 )	F		25		1	710.4	234.3	1,338.8	<DL	6	26	19-35
S424_06 ( 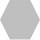 )	F		36		1	392.3	224.5	1,154.9	<DL	4	43	34-67
S363_06 ( 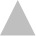 )	M		32		1	696.1	180.6	1,503.0	<DL	6	6	3-13

**Figure 5:**
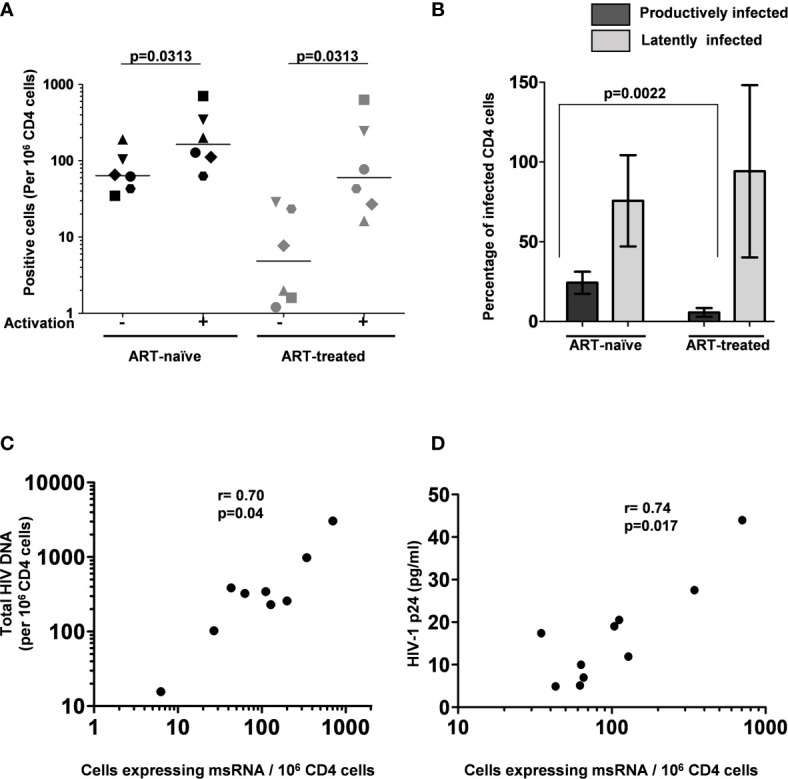
The frequency of persistently infected cells in donor blood samples. **(A)** The frequency of CD4^+^ T cells producing msRNA spontaneously (No activation) or after 12 h of stimulation (activation) in ART-naïve (n=6) and ART-treated (n=6) participants. Each symbol represents a donor. The horizontal bars represent the median values of the positive cell frequency. Wilcoxon matched-pairs signed-rank test was used for statistical analysis. **(B)** Histograms depicting the percentage of productively and latently infected CD4^+^ T cells in ART-naïve and ART-treated participants (mean ± SEM). P-value was obtained from the Mann–Whitney test. **(C)** Correlation between msRNA-producing CD4^+^ cell number and total HIV-1 DNA copies. Genomic DNA extracted from CD4^+^ cells of nine study participants was used to quantitate the total HIV-1 DNA load using a ddPCR format as described in materials and methods. In parallel, the number of CD4^+^ cells expressing viral transcripts was evaluated using the U-TILDA format. Each dot represents a study participant. Spearman’s rank correlation analysis was performed to estimate the correlation. **(D)** Correlation between msRNA-producing CD4^+^ cell number and p24 production from the cells. Each dot represents one of the nine study participants used in the assay. The number of CD4^+^ cells expressing viral transcripts was determined as in panel **(C)** The amount of p24 secreted into the medium by one million enriched CD4^+^ cells after three days following the activation with PMA (100 ng/ml) and Ionomycin (1 µg/ml) was determined using a commercial kit. Spearman’s rank correlation analysis was performed to estimate the correlation.

PVL, Plasma viral load; DL, Detection limit. The symbols within parenthesis identify participants as in **Figure 5**.

The frequency of CD4^+^ T cells expressing HIV-1 transcripts with and without activation was determined using the optimized protocol described in Material and Methods. The data ascertain that the frequency of the cells producing early viral transcripts enhanced significantly in both the groups following activation (**Figure 5**). The median number of transcript-producing cells per million CD4^+^ cells increased from 63.7 to 164.0 and 4.8 to 60.0 in the ART-naïve and ART-exposed groups, respectively (**Figure 5A**). While the enhanced transcript induction in a group was significant, that of individual subjects varied considerably. For example, in the CD4^+^ cells of participant S338_02 under the ART-naïve group, 34.7 and 707.7 cells produced viral transcripts before and after activation, respectively, demonstrating a 20-fold enhancement approximately. Whereas in the CD4^+^ cells of participant S137_02 belonging to the same group, there was not even a two-fold enhancement in the induction of viral transcripts, with 43.0 cells increasing to 63.0 cells following activation. A certain percentage of cells among the CD4^+^ cells of both the groups produced early viral transcripts spontaneously without activation (**Figure 5B**). While 24.3 ± 6.9% of cells spontaneously produced multiply spliced RNA in the ART-naïve group, only 5.8 ± 2.8% of cells demonstrated productive infection in the ART-exposed group in the absence of activation. Following activation, 75.7 ± 28.6 and 94.1 ± 54.0% cells produced viral transcripts representing approximately 3- or 10-fold enhancement in induction in the ART-naïve and ART-exposed groups, respectively. **Table 3** shows the number of cells expressing multiply spliced RNA per million CD4^+^ cells and the 95% confidence interval for the measurements.

Additionally, several publications previously demonstrated a positive correlation between the total HIV-1 DNA copy number and TILDA values (22, 25). Using negatively enriched CD4^+^ cells of nine study participants, we also found a statistically significant correlation between HIV-1 DNA and msRNA levels using Spearman’s rank correlation analysis and found a significant level of correlation (ρ = 0.70 and p = 0.04, **Figure 5C**). We found a similar statistically significant correlation between the number of cells expressing msRNA and the p24 antigen secreted into the medium (ρ = 0.74 and p = 0.017, **Figure 5D**). In summary, the U-TILDA captured the activation kinetics of the HIV-1 latent reservoirs in total CD4^+^ cells of ART-naive and ART-exposed participants.

## Discussion

The precise quantitation of the latent reservoir size and monitoring latency reversal kinetics need an experimental strategy characterized by high detection sensitivity and breadth of detection. Various experimental strategies have been reported, each endowed with its own merits and limitations (30). An ideal assay that can satisfy all the requirements is elusive for several technical challenges, including the extremely low frequency of infected cells, a further low frequency of cells harboring replication-competent proviruses, an even smaller number of replication-competent viruses getting induced under activation of any kind, and the immense genetic diversity of the HIV-1 genetic subtypes.

Despite the specific limitations, TILDA offers several technical advantages; therefore, it presents an ideal format, best-suited to examine and interrogate HIV-1 latency and latent reservoir. One technical limitation of TILDA is its inability to detect uninduced, replication-competent proviruses. Notwithstanding this limitation, TILDA is endowed with many technical merits, including its ability to exploit the PCR amplification advantage to detect viral transcripts, the need for a small size of a blood sample, and the short duration of the assay format. Here, we successfully circumvented one significant technical limitation of the TILDA by targeting highly conserved sequences of exons 1 and 7, we enhanced the breadth of detection of the assay. Exon-1 is one of the highest conserved regions of the viral genome (31). By positioning the two forward primers and the probe, we have circumvented the severe limitation of genetic variation of the Tat exons (**Figure 2**). Likewise, the sequences selected to position the two reverse primers have also been highly conserved. Because of the high sequence conservation, we could amplify all the eight molecular clones representing five major HIV-1 subtypes with comparable efficiency (**Figure 4**). These five major HIV-1 subtypes (A, B, C, D, and E) represent approximately 77% of global infections among themselves (32). Although we could not test other HIV-1 subtypes, due to the non-availability of infectious molecular clones, given the high-level sequence conservation, the primers described here are best suited for TILDA with the highest sensitivity and breadth of detection.

Despite the advantages offered by TILDA, there is still scope for improvements. Pezzi et al. recently demonstrated that RNA isolation using a bead-based method to remove other impurities from the cell lysate could enhance assay sensitivity (33). However, the direct use of cell lysate in TILDA minimizes sample loss and simplifies the assay protocol. Nevertheless, a limit exists that adding more cells to the reaction compromises the assay efficiency. The use of polymerases in TILDA that are intrinsically resistant to sample impurities can be one possible solution (34). In our protocol, we used the cell lysate directly without storing the samples to prevent the loss of sensitivity of TILDA, as suggested by Châtel et al. (35). One technical limitation of the experimental strategy described here is the longer sizes of the amplified fragments. Amplification sensitivity is inversely associated with the size of the fragment being amplified typically. However, we demonstrated that despite the amplification of relatively larger fragments, there was no compromise in the detection sensitivity of the assay (**Figure 3**). We could not evaluate any primary clinical viral samples of non-HIV-1C given the scarcity of such infections in India. In summary, the modified TILDA reported here should help evaluate HIV-1 latent reservoirs with greater efficiency.

## Data Availability Statement

The original contributions presented in the study are included in the article/supplementary material. Further inquiries can be directed to the corresponding author.

## Ethics Statement

The studies involving human participants were reviewed and approved by The Human Ethics and Biosafety Committee of Jawaharlal Nehru Centre for Advanced Scientific Research (JNCASR), Bangalore. The patients/participants provided their written informed consent to participate in this study.

## Author Contributions

KM performed research and wrote the original draft. YG, SM, AD’S, AA, DS, NP, and NN performed research. UR and GS designed research, reviewed, and edited the article. All authors contributed to the article and approved the submitted version.

## Funding

This work was supported by funds from the Department of Biotechnology, Ministry of Science and Technology, Government of India (Sanction order no. BT/IN/Netherlands/RG/40/2015) to UR and from Rajiv Gandhi University of Health Sciences, State Government of Karnataka, India (Sanction order no. RGU/Adv. Res/CR/04/2018-19) to both UR and GS.

## Conflict of Interest

The authors declare that the research was conducted in the absence of any commercial or financial relationships that could be construed as a potential conflict of interest.

## Publisher’s Note

All claims expressed in this article are solely those of the authors and do not necessarily represent those of their affiliated organizations, or those of the publisher, the editors and the reviewers. Any product that may be evaluated in this article, or claim that may be made by its manufacturer, is not guaranteed or endorsed by the publisher.
